# Vulnerability mapping as a tool to manage the environmental impacts of oil and gas extraction

**DOI:** 10.1098/rsos.171044

**Published:** 2017-11-29

**Authors:** Surina Esterhuyse, Frank Sokolic, Nola Redelinghuys, Marinda Avenant, Andrzej Kijko, Jan Glazewski, Lisa Plit, Marthie Kemp, Ansie Smit, A. Tascha Vos, Michael J. von Maltitz

**Affiliations:** 1 Centre for Environmental Management, University of the Free State, PO Box 339, Bloemfontein, South Africa; 2 Department of Sociology, University of the Free State, PO Box 339, Bloemfontein, South Africa; 3 Department of Mathematical Statistics and Actuarial Science, University of the Free State, PO Box 339, Bloemfontein, South Africa; 4 Department of Geology, University of Pretoria Natural Hazard Centre, Africa, University of Pretoria, Private Bag X20, Hatfield, Pretoria 0028, South Africa; 5 Institute of Marine and Environmental Law, University of Cape Town, Private Bag X3, Cape Town, South Africa

**Keywords:** vulnerability map, South Africa, unconventional oil and gas, regulation, environmental assessment and protection

## Abstract

Various biophysical and socio-economic impacts may be associated with unconventional oil and gas (UOG) extraction. A vulnerability map may assist governments during environmental assessments, spatial planning and the regulation of UOG extraction, as well as decision-making around UOG extraction in fragile areas. A regional interactive vulnerability map was developed for UOG extraction in South Africa. This map covers groundwater, surface water, vegetation, socio-economics and seismicity as mapping themes, based on impacts that may emanate from UOG extraction. The mapping themes were developed using a normative approach, where expert input during the identification and classification of vulnerability indicators may increase the acceptability of the resultant map. This article describes the development of the interactive vulnerability map for South Africa, where UOG extraction is not yet allowed and where regulations are still being developed to manage this activity. The importance and policy implications of using vulnerability maps for managing UOG extraction impacts in countries where UOG extraction is planned are highlighted in this article.

## 1. Introduction

Unconventional oil and gas (UOG) extraction, its related impacts and the management of this activity to ensure environmental protection, is a controversial issue in many countries worldwide. Various biophysical and socio-economic impacts, ranging from positive to negative, may be associated with UOG extraction. Vulnerability maps that show the location of vulnerable entities are useful tools that may assist governments in their decisions to allow, or not allow, UOG extraction in certain fragile areas, and may aid in the regulation of UOG extraction in ecologically sensitive areas.

Internationally, the importance of vulnerability maps is increasingly being realized. Russack [1] developed a vulnerability map for Allegheny County, Pennsylvania, and Rivard *et al.* [2] indicated areas where regional overview aquifer studies have been performed in Canada related to UOG extraction in an overview of Canadian shale gas production. These maps focused on specific counties and shale formations, and identified vulnerability to water contamination from fracking operations, which is the aspect of most concern when one considers water requirements by fracking [3–5] and possible freshwater contamination [3,6,7]. While vulnerability mapping of natural resources is important for its protection, mapping socio-economic vulnerabilities is also important for proper spatial planning and infrastructure development in areas where UOG extraction is planned or practised. In this respect, Ogneva-Himmelberger & Huang [8] identified the spatial distribution of unconventional gas wells and vulnerable human populations in the Marcellus shale formation.

It is, however, important to note that all of the above-mentioned maps have been produced retrospectively, after UOG operations have already started, and do not represent baseline maps. Other maps of note for the management of UOG impacts include the US database and interactive map viewer called ‘Fracktracker’ [9] where information such as well localities, spills, pipelines, shale deposits, etc. have been loaded as overlays with base map layers being predominantly topographic, satellite imagery or ‘OpenStreetMap’, and provides a real-time management option for regulators. These interactive maps show the location of current UOG infrastructure, but do not contain base maps that indicate vulnerability for specific entities.

The importance of developing baseline maps for proper natural resource protection relates to the fact that baseline monitoring could provide direct input into an adaptive management approach by reducing uncertainty in our understanding of the effects of shale gas development and risks to natural resources and humans by performing spatial analyses, vulnerability assessments and threshold and toxicity evaluations, among others [10]. Baseline monitoring and mapping would also provide an impartial scientific base to support the sustainable use of resources in shale gas development areas and state various data shortcomings that hamper proper natural resource protection. Countries that are still planning to embark on UOG extraction thus have the unique opportunity to address data needs and perform crucial baseline vulnerability mapping before UOG extraction starts.

This paper discusses the development of a South African UOG vulnerability map that covers the whole country, in a project that was performed for the South African Water Research Commission [11]. No UOG resources are currently being extracted in South Africa, but may become a possibility in the future. This map thus represents baseline sensitivity of certain aspects of the biophysical and socio-economic environments. It focuses on five mapping themes: surface water, groundwater, vegetation, seismicity and socio-economic aspects. These mapping themes have been based on the most important impacts from UOG extraction on resources [11]. The map (https://fracking.webmaps.co.za/map.php) displays regional scale South African data that may indicate vulnerable localities for these mapping themes. It also includes various base maps and overlays to assist regulators in performing a more thorough assessment of vulnerability. This map was developed in a way that would specifically assist regulators in assessing vulnerability on a larger spatial scale by considering the possible effects of UOG development across provincial boundaries and themes (e.g. they can compare vulnerability of groundwater versus socio-economic factors for a specific region). Although the South African vulnerability map does not include information on drilling sites yet (as UOG has not been developed here yet), it has been used during the strategic environmental assessment that was performed for the South African Department of Environmental Affairs [12]. The importance and use of vulnerability maps for planning purposes in countries that plan to embark on UOG extraction is explained, by discussing specifically the development of the water and socio-economic mapping themes.

## 2. Methods

A multi-disciplinary team (various water specialists, vegetation specialists, socio-economists, seismicity experts and legal experts) developed a vulnerability map specifically for UOG extraction in South Africa. Vulnerability mapping usually entails the mapping of exposure, sensitivity and coping capacity indicators [13–15]. The greater the exposure or sensitivity of a system, the greater the vulnerability, and conversely, the greater the coping capacity, the less vulnerable the system will be. Classically, only sensitivity indicators are mapped for biophysical systems [16]. The ‘impacts’ method [17] was therefore used to identify sensitivity indicators for this map. The team decided to concentrate on sensitivity indicators as related to the exposure to UOG extraction, because variables for these elements are most often and easily investigated and quantified. Schauser *et al*. [16] also reported that recognition and classification of indicators into exposure, sensitivity and coping/adaptive capacity is often too complex to yield usable results.

A normative approach was followed where experts had to identify sensitivity indicators for the vulnerability map. Although this approach requires time and resources and is limited in its application and transferability to other regions (e.g. other countries), the integration of expert knowledge provides support for the weighing and aggregation of the indicator components, and may increase the acceptability of the results. It is also widely acknowledged that the involvement of stakeholders in the development of indicators is key to identifying relevant vulnerability indicators [13,18].

Key informants related to each discipline were identified during the indicator identification phase with the aim of using them throughout the mapping exercise. It was deemed important to use experts, in order to ensure proper adherence to policy goals, and also encourage transparency, credibility and pragmatism. The experts who were chosen to participate in the study as key informants had to have knowledge of UOG extraction by means of hydraulic fracturing, or had to be involved in research related thereto. In some cases, not all the participants could comply with this requirement, and this highlights how new this field of research is in South Africa. However, experts still needed to be consulted for the indicator identification phase, as the contextualization and information given to the experts could assist with identifying relevant indicators. The team implemented the ‘issues/impacts concept’ [13,19] during the indicator identification phase by giving experts information on the possible impacts or issues that may emanate from exposure to UOG extraction by means of hydraulic fracturing. Possible positive and negative impacts during each of the UOG extraction phases (exploration, extraction and post-extraction) for the mapping themes were identified by team members during the execution of the background review, and are based on the literature. The information on potential impacts helped to guide the development of indicators, as well as an understanding of the links between indicators [20]. It also helped to clarify the rationale for the selection of specific indicators. Experts could therefore assess the usefulness and appropriateness of different proposed sensitivity indicators.

**Figure 1.**
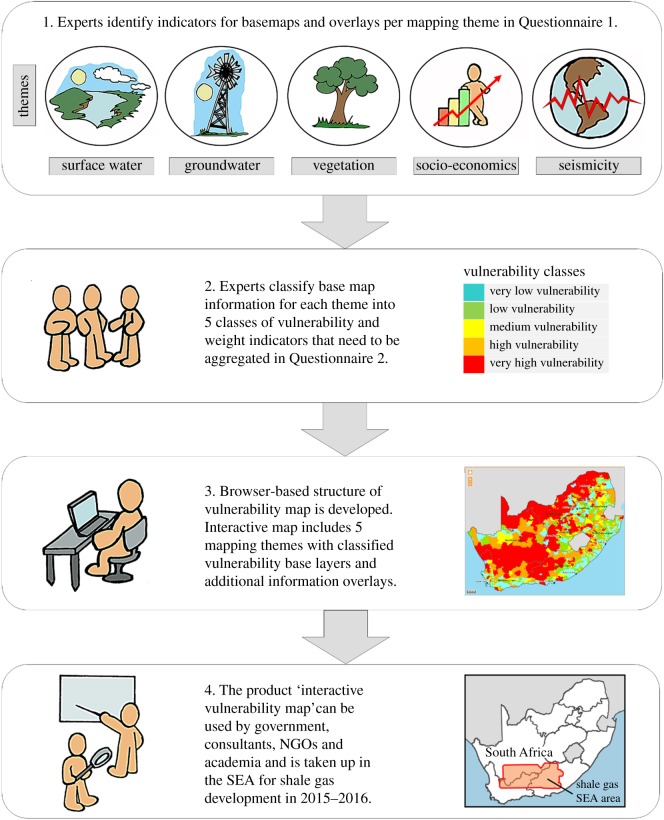
Process for the development of the interactive vulnerability map.

Two questionnaires were used during the mapping process to engage with the experts for identifying indicators, classifying the base layer vulnerabilities and identifying important additional overlay information. The process followed for vulnerability mapping is summarized in figure 1.

During ‘Questionnaire 1’, the exploratory indicator identification questionnaire, experts in each discipline were asked to review the appropriateness (on a scale from 1 to 10) of possible vulnerability indicators, to give reasons for their answers and to suggest additional indicators where applicable. These answers were used to identify useful sensitivity indicators that can be used for vulnerability mapping of each aspect. During the indicator identification phase, experts were also asked to indicate data availability if they indicated the use of alternative indicators.

Expert feedback from ‘Questionnaire 1’ was used to identify the relevant indicators that were to be classified and used as base layers in the different vulnerability mapping themes and indicators that were to be flagged and used as overlay indicators. Overlay indicators convey important additional information but do not comply with the requirements of serving as a base layer (for instance, it does not cover the whole of South Africa). Areas where legislation prescribes how certain activities may take place, where activities are prohibited by law or where assessment or protection zones are required have also been mapped as overlay indicators. For seismicity, only one indicator (seismic hazard) was used and questionnaires have thus not been developed for seismicity.

Base layer indicators of each mapping theme were classified into five classes of vulnerability (very low, low, medium, high and very high), and weighted in instances where indicators were to be aggregated. Weights express the contribution and relative importance of the individual indicator component. Indicator weights were determined by means of the budget allocation method [18] to ensure consistency between mapping approaches of the different mapping themes. The budget allocation method is a participatory method in which experts are given a ‘budget’ of *N* points, which must be distributed over a number of indicators, ‘paying’ more for those indicators whose importance they want to stress. Apart from ensuring consistency between mapping approaches, this method also makes re-weighting of mapping components and disaggregation of indicators easier. The classification and weighting was done via a second round of questionnaires to the experts (figure 1). Overlay indicators did not require weighting and were included as overlays on the base maps in the browser.

For indicator aggregation, various studies recommend that aggregation methods be as simple as possible, in order to ensure transparency and allow disaggregation of indicators [17]. Independent indicators were aggregated by means of the simple additive weighting method and dependent indicators were aggregated by using the weighted product method [21]. Interaction between different indicators that were to be aggregated for each aspect was investigated by the mathematical statistician, using various statistical methods, including multiple correspondence analysis (MCA) and principal component analysis (PCA).

The team decided that the seismicity, groundwater, surface water, vegetation and socio-economic mapping themes would not be aggregated for the interactive vulnerability map, because the usefulness of such an aggregated map may be limited. This is because different South African government departments have mandates to manage different components, e.g. the Department of Water and Sanitation (DWS) must protect, manage and monitor groundwater and surface water resources, while the Department of Environmental Affairs (DEA) is responsible for the protection of vegetation and biodiversity. However, the base layer indicators for the socio-economics mapping theme and the vegetation mapping theme were aggregated and included on the interactive map together with the separate base layers.

## 3. Results and discussion

The mapping process used 43 external experts in total. A list showing the expert usage for each aspect is given in table 1. Some respondents were not prepared to contribute inputs to the study, due to the sensitivity of the UOG extraction issue, or the lack of sufficient knowledge on the issue.

**Table 1.** Experts used during the development of the UOG vulnerability map.

respondent information	surface water	groundwater	vegetation	socio-economics
number of respondents approached	20	14	10	14
number of informants participating	10	12	10	11
profile of respondents	invertebrate specialists; vertebrate specialists; water quality specialists in academia, government and industry	groundwater specialists in academia, consultancy and government	vegetation specialists in consultancy and academia	academia; agricultural economists; environmental consultants; human geographers; population–environment–development (PED) specialists

Owing to the sensitive and politicized nature of UOG extraction in South Africa, and in order to adhere to ethical research practices, all responses were treated anonymously.

‘Base maps’ that indicate vulnerability were divided into five classes (very low, low, medium, high and very high). The range of values for each class of vulnerability was defined by the experts individually for each indicator. ‘Overlay maps’ were not classified, but indicate important additional information, e.g. critical biodiversity areas that have been identified in certain parts of the country, but for which regional scale data do not yet exist. The vulnerability map is browser-based and the user can zoom, pan and click on features to obtain more information on a specific feature within a mapping theme. The mapping themes are indicated separately and the user can switch between the different mapping themes.

The base layer indicators and overlay indicators that were selected for each mapping theme are presented in table 2. Most of the themes have their theme-specific base layer and overlay indicators (identified by the experts), but some themes share overlay indicators that are important to more than one mapping theme. For example, ‘rivers’ and ‘water management’ areas are important to both the surface water and groundwater mapping themes. Similarly, ‘geological structures' are important to both the groundwater and seismicity mapping themes. The overlay layers ‘mining and petroleum resource production legally prohibited’, ‘onshore PASA permit areas (technical cooperation permit (TCP) and exploration right (ER) areas)’ and ‘roads’ were included as an overlay on all the mapping themes, as it is important to identify areas where oil and gas production is planned or is prohibited, irrespective of the mapping theme, while ‘roads’ enables the regulator to identify the locality on the map, irrespective of the mapping theme.

**Table 2.** Base layer and overlay indicators.

indicators	surface water	groundwater	seismicity	socio-economics	vegetation
base layer indicators	river condition/vulnerability (figure 2*a*)	drastic groundwater vulnerability	peak ground acceleration	aggregated map (figure 3*f*)	aggregated map
				population indicators: population density per area (figure 3*a*); % of population under 5 years of age per area (figure 3*b*)	ecosystem threat status
	wetland vulnerability (figure 2*b*)			environment indicators: % of population dependent on groundwater per area (figure 3*c*)	ecosystem protection level
				development indicators: % of population employed by agriculture per area (figure 3*d*); % of female-headed households per area (figure 3*e*)	
overlay indicators	wetland clusters	Vegter's groundwater regions	population density	assessment in areas as identified in regulations under the *Astronomy Geographic Advantage Act*	aquifer-dependent ecosystems
	threatened and near threatened fish species	subterranean groundwater control areas		subterranean groundwater control areas	critical biodiversity areas and associated ecological support areas
	strategic water sources	boreholes			vegetation of South Africa
	rivers				
	water management areas				
		geological structures (1 : 1 000 000 scale) and springs			
	mining and petroleum resource production legally prohibited				
	onshore PASA permit areas (TCP and ER areas)				
	roads				

Examples and a short discussion of the surface water mapping theme (to illustrate a biodiversity aspect) and the socio-economic mapping theme (to illustrate the socio-economic aspect) will be presented in §§3.1–3.2. For full details on the mapping of all the themes, see Esterhuyse *et al*. [11].

### 3.1 Surface water mapping theme

#### 3.1.1. Base maps

Two indicators, namely, ‘river condition/vulnerability’ using the default ecological category (DEC) data from the Present Ecological State and Ecological Importance and Sensitivity (PESEIS) study [22], and ‘wetland vulnerability’ using National Freshwater Ecosystem Priority Areas (NFEPA) wetland rank data [23], were used as base map indicators for the surface water vulnerability map.

After identification of vulnerability map indicators for the surface water theme, a second questionnaire was sent to the experts, who assessed the classification of the levels of vulnerability for each indicator, confidence in the data to be used and the weighting of the individual indicators in the final surface water vulnerability map.

For *indicator 1 (river condition)*, ‘uncategorized’ includes all subquaternary reaches (SQRs) where no DEC could be determined due to the episodic nature of the small tributaries (no data are available for these SQRs; they are mostly reaches where stream flow is absent for very long periods and they are often little more than drainage lines). The DWS has a policy not to manage any river as a class E (seriously modified) or F (critically modified) as this is considered to be unsustainable (hence no such class has been identified during the DEC classification). The DEC indicates how vulnerable even the impacted reaches would be to additional impacts. Each SQR was classified in terms of vulnerability and colour coded according to the suggested vulnerability classes (table 3).

**Table 3.** Vulnerability classes for river condition using the DEC from the 2011–2013 PESEIS data [22] and for wetland ranks, using the wetland rank data from the NFEPA study [24].

river condition			wetland ranks		
vulnerability description	suggested classes	colour code	vulnerability description	suggested classes	colour code
uncategorized	DEC = uncategorized	grey	not applicable		
very low	DEC = E/F (E, seriously modified; F, critically modified)	blue	very low	no wetland	blue
low	DEC = D (largely modified)	green	low	not WetFEPA	green
moderate	DEC = C (moderately modified)	yellow	moderate	presence of frogs and or CWAC (coordinated waterbird counts)	yellow
high	DEC = B (largely natural/few modifications)	orange	high	presence of cranes	orange
very high	DEC = A (unmodified/natural)	red	very high	presence of WetFEPA and/or Ramsar site	red

For *indicator 2 (wetland ranks)*, wetlands were ranked according to the NFEPA study [24] at the level of a wetland unit (entire wetland system and could comprise several wetland ecosystem types or wetland conditions). Subnational biodiversity priority data were used to identify important wetlands. Ramsar sites and wetlands supporting threatened frog, water bird and crane species were identified and included in the vulnerability map. Wetlands that formed a group of more than three wetlands within 1 km (wetland cluster) were also included and expert opinion was used to verify important wetlands [23].

Experts indicated that wetlands identified as WetFEPAs should also be included with Ramsar wetlands as being of very high vulnerability/ranking. In the NFEPA study, a politically acceptable national biodiversity target for South Africa's freshwater ecosystems was to maintain at least 20% of each major freshwater ecosystem type in a good condition. The identified WetFEPAs therefore only represent the target of 20% of wetland types to be protected and if these WetFEPAs were to be impacted on further, the biodiversity target set in the NFEPA study would not be reached [23]. FEPA wetlands were buffered using a 1 km buffer. The FEPA wetlands are considered to be of the highest biodiversity importance.

The classification of indicator 1 (river condition/vulnerability) and indicator 2 (wetland ranks) can be seen in table 3.

**Figure 2.**
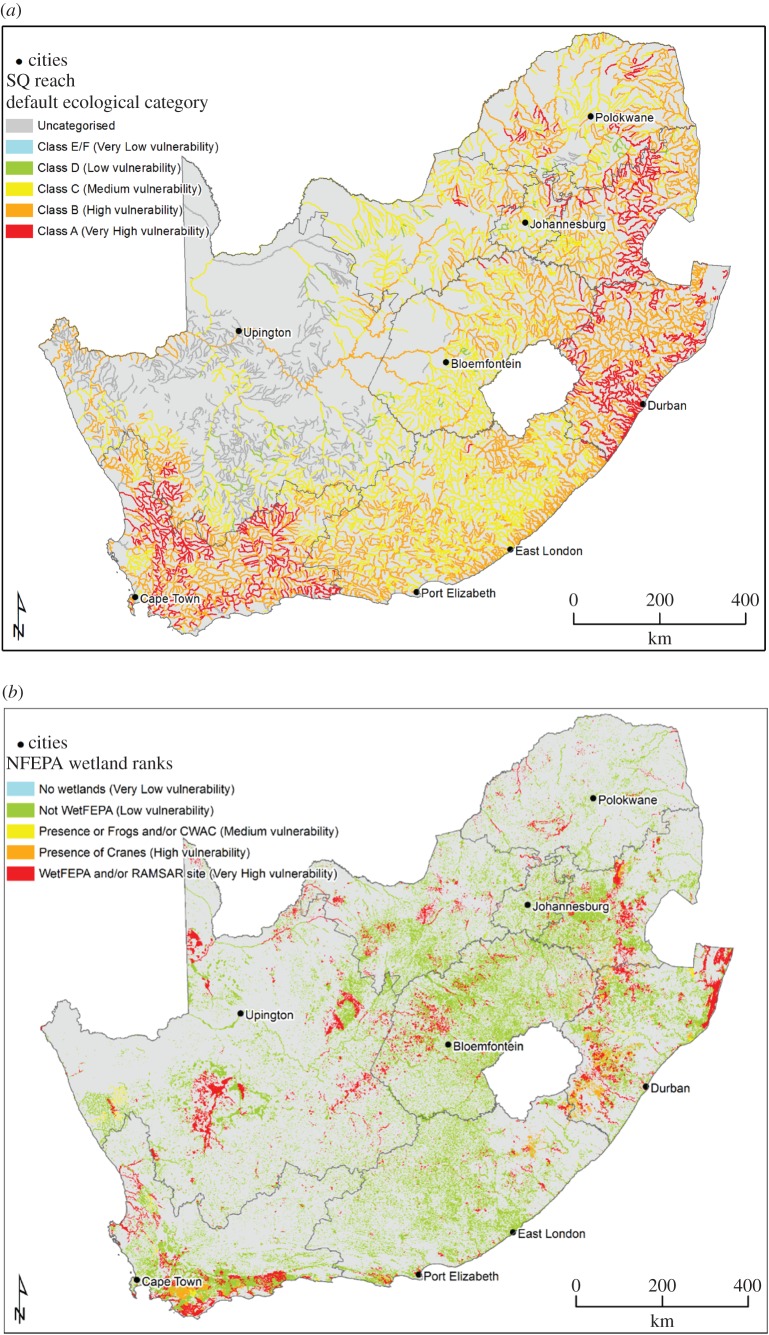
(*a*) Map indicating river condition/vulnerability using DEC data from the 2011–2013 PESEIS data [22]. (*b*) Wetland vulnerability according to five vulnerability classes using wetland rank data from the NFEPA study [22].

Figure 2*a* shows the river condition for South Africa and figure 2*b* the wetland ranks for South Africa. In figure 2*a*, it is clear that the northwestern part of South Africa has a large amount of uncategorized rivers. These uncategorized rivers/tributaries would need additional local scale research, in a future study, to determine their actual vulnerability and it should not be assumed that they are not vulnerable to UOG impacts. Figure 2*b* shows that wetlands with very high vulnerability occur across South Africa, making wetland protection an important aspect during UOG extraction.

#### 3.1.2. Overlay maps

The following overlay indicators were included on the surface water vulnerability base map: ‘wetland clusters’ and ‘critically endangered, endangered, near threatened, vulnerable, least concerned, and data deficient fish species'. These indicate the areas that require caution and where more detailed studies are recommended before UOG extraction can be considered.

The raw data from the Fishsanc-All species database from the NFEPA study [23,24] were included as the ‘critically endangered’, ‘endangered’, ‘near threatened’, ‘vulnerable’ and ‘least concerned’ fish species in each subquaternary catchment. Species identified by the IUCN [25] as data deficient, but deemed important by specialists in the NFEPA study, were also included as ‘data deficient’. Protecting these areas would keep further freshwater species from becoming threatened and would prevent those fish species that are already threatened from becoming extinct.

### 3.2 Socio-economic mapping theme

#### 3.2.1. Base maps

The population–environment–development (PED) nexus framework, an accepted analysis framework for analysing people–environment interactions, both internationally and nationally [26,27], formed the analytical basis of indicator selection for the socio-economic mapping theme. This framework assured that indicators are systematically selected to reflect the various facets of the social environment, which include population, environment (specifically pertaining to the linkages between human health and the environment) and development. The selected socio-economic indicators, based on expert input, can be seen in table 4.

**Table 4.** Base layer indicators per mapping dimension of the PED nexus.

mapping dimension	base layer indicator
population	population density per area
	% of population under 5 years of age per area
environment	% of population dependent on groundwater per area
development	% of population employed by agriculture per area
	% of female-headed households per area

Emphasis was placed on selecting indicators that would adequately reflect how those populations that are already considered vulnerable would be affected by the negative impacts of UOG extraction. These groups are, among others, the poor, women, children and ethnic minorities [28]. The indicator ‘% of population under 5 years of age’ describes a part of the population that is more vulnerable to adverse environmental impacts. This indicator is linked specifically to groundwater and air pollution, because respiratory diseases and water-borne diseases are among the main causes of death in children under 5. The health status of children is also an accepted indicator to gauge the overall health status of populations. The indicator ‘% of female-headed households’ is directly informative of social and economic vulnerability in poverty-stricken areas. Areas with high numbers of female-headed households may be more vulnerable to UOG extraction impacts, due to women being more vulnerable to rising economic inequality, the spread of HIV and increased social ills that are brought about by the influx of money and workers into an area. Lastly, the percentage of the population employed by the agricultural sector may serve as a proxy indicator for economic trends in the agricultural sector, e.g. a drop in employment rates might point to an exodus of commercial farmers due to changing or hostile farming conditions, such as a decline in available groundwater, or an increase in polluted water. Agriculture employs large numbers of unskilled and semi-skilled people who will not be absorbed into the shale gas sector when mining impacts on agricultural productivity, because their skills are not compatible with the employment requirements for UOG extraction.

After identification of vulnerability map indicators during a first round of questionnaires, a second questionnaire was sent to the experts, who then assessed the classification of the levels of vulnerability for each indicator, confidence in the data to be used and the weighting of the individual indicators in the final socio-economic vulnerability map. A preliminary classification was presented to nine of the 11 experts who agreed to contribute their inputs to the vulnerability classification. Based on the results of this questionnaire, the vulnerability classifications for the different base layer indicators can be seen in table 5.

**Table 5.** Vulnerability classification of base layer socio-economic indicators.

		indicator				
vulnerability description	colour code	number of people per km^2^	% of children under 5 per area	% of population dependent on groundwater as a domestic water source	% of the population employed by agriculture per area	% of female-headed households per area
very low vulnerability	blue	0–10	<11.49	0–10%	0–1.99%	<36%
low vulnerability	green	11–50	11.5–12.49	11–20%	2–3.99%	37–40%
medium vulnerability	yellow	51–100	12.5–13.99	21–30%	4–7.99%	41–45%
high vulnerability	orange	101–500	14–15.49	31–50%	8–15.99%	46–50%
very high vulnerability	red	>500	≥15.5	≥51%	≥16%	≥51%

These base layers have been aggregated multiplicatively into an aggregated map for socio-economic vulnerability because scatterplots, MCA and PCA showed interaction between the different indicators. Scatterplots indicated that the most obvious relationship is present in the population density and groundwater dependence plot, with wards more dependent on groundwater exhibiting lower population density (but not necessarily vice versa).

A budget allocation weighting approach was followed in the weighting of the indicators for aggregation. Exploratory interviews were also conducted with key informants to confirm the weighting of the indicators. The weighting that was applied for the aggregated map can be seen in table 6.

**Table 6.** Socio-economic theme base layer weighting percentages.

indicator	weight
population density	5%
children under 5 years	15%
groundwater dependence	40%
female-headed households	10%
employment	30%

**Figure 3.**
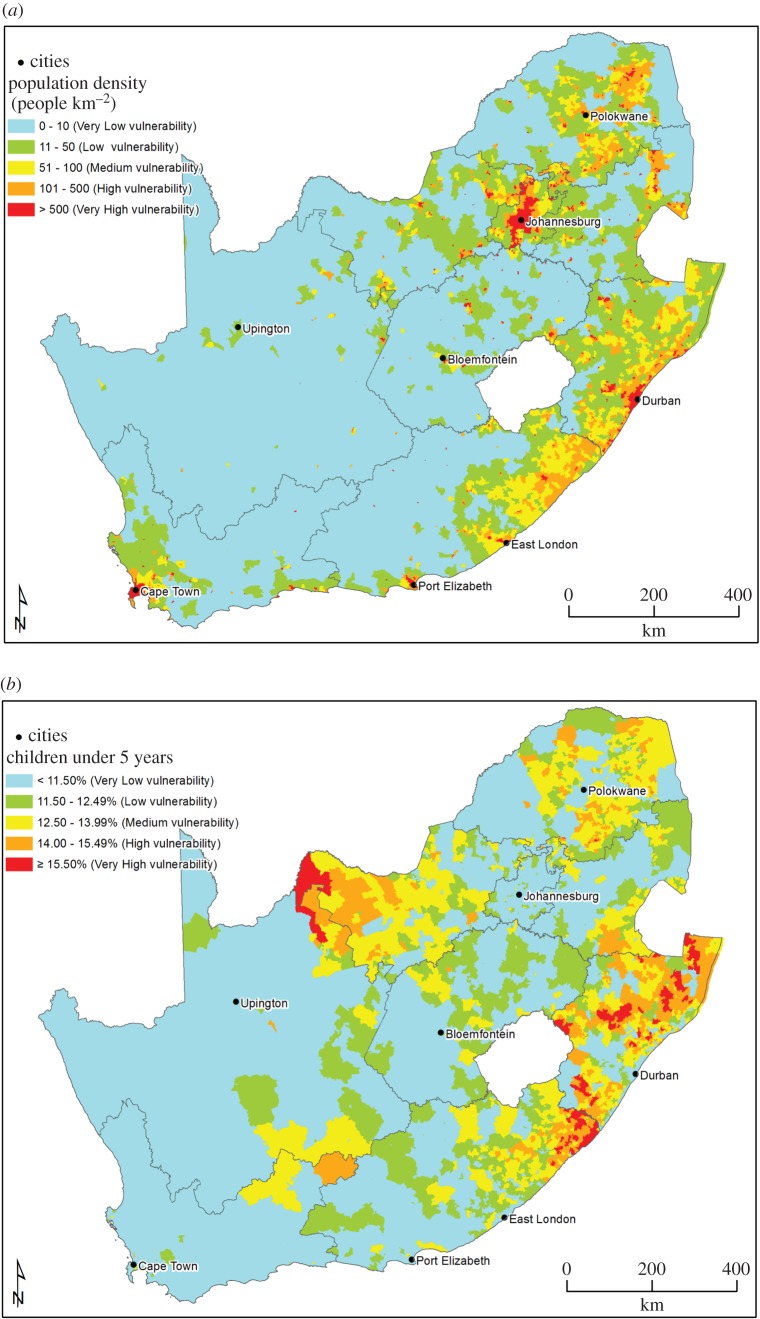

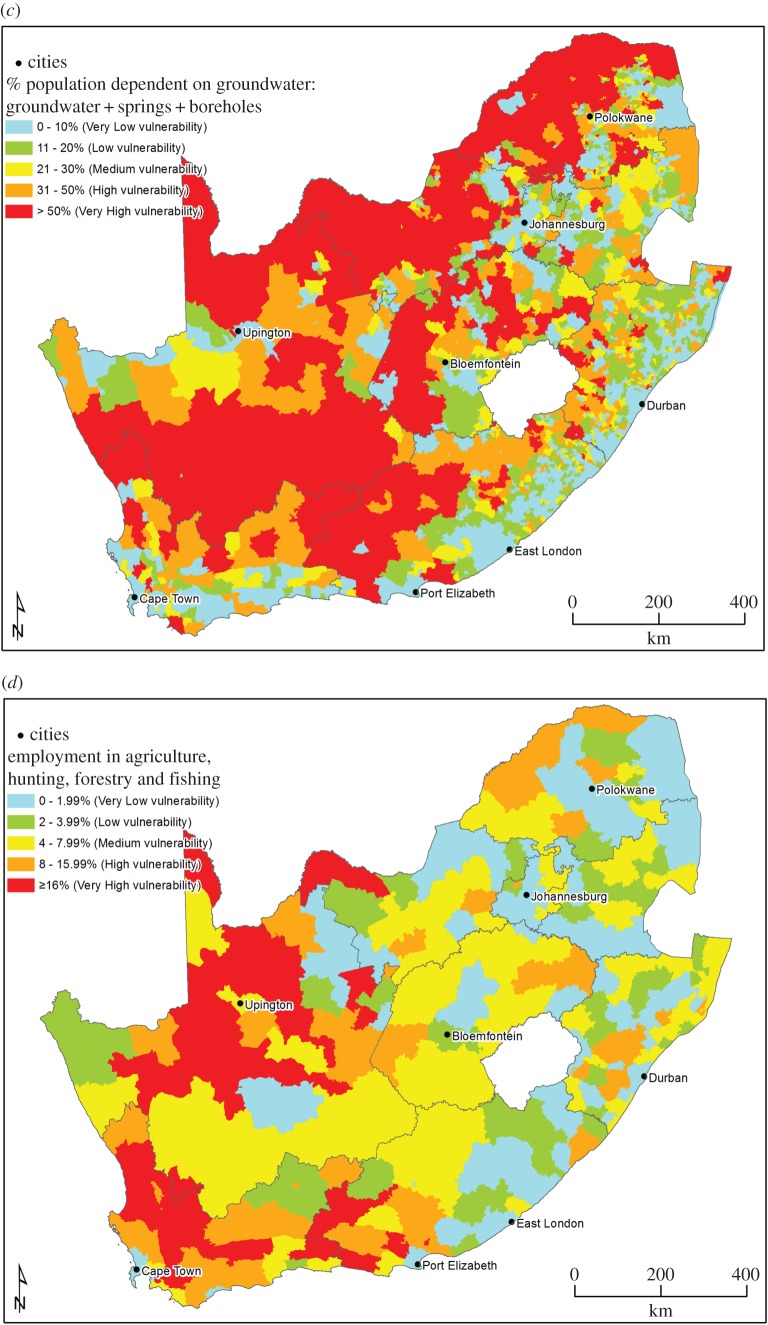

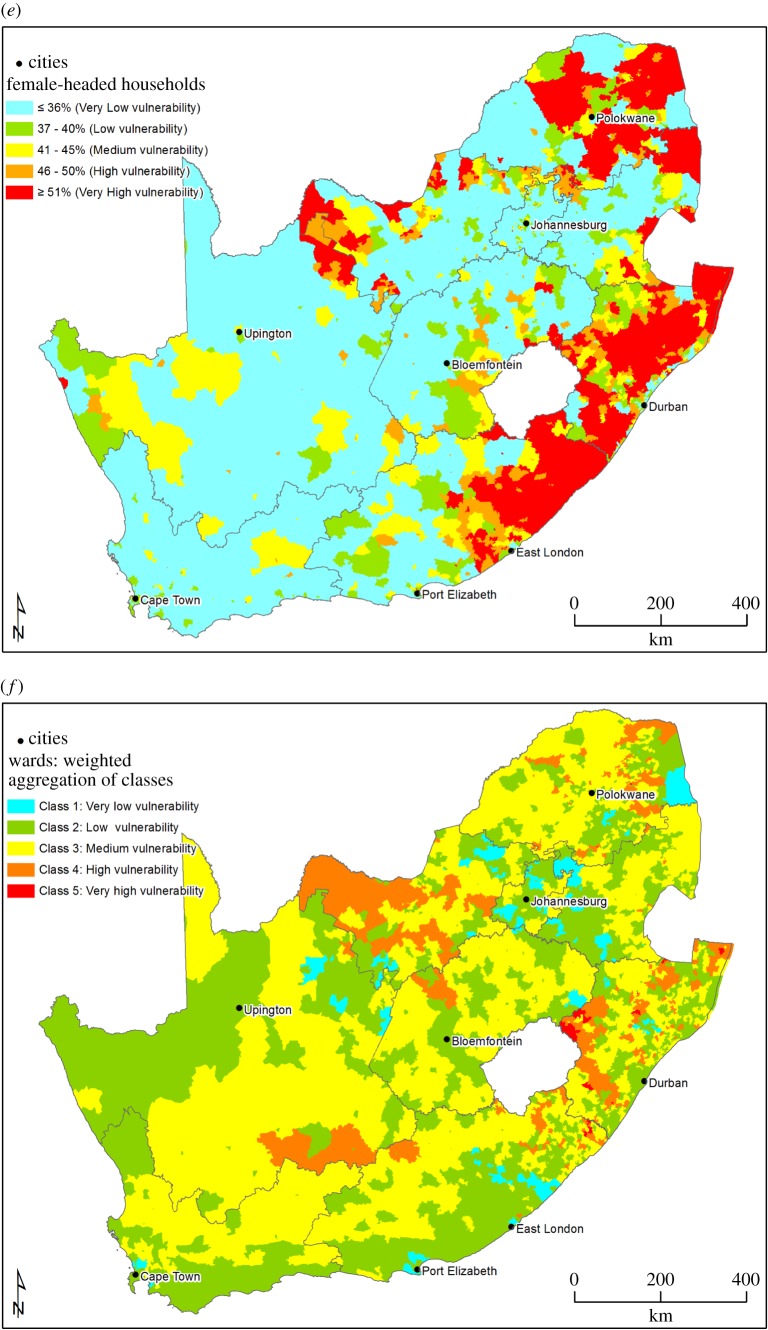
(*a*) Population density—data sources [29,30]. (*b*) Percentage of children under 5 years per area—data sources [29,30]. (*c*) Groundwater dependence for domestic use per area—data sources [29,30]. (*d*) Percentage of people employed by agriculture per area—data sources [29,30]. (*e*) Percentage of female-headed households per area—data sources [29,30]. (*f*) Aggregated map for socio-economics area—data sources [29,30].

Figure 3*a*–*e* shows the base layers for the socio-economic mapping theme, while figure 3*f* shows the aggregated base map for socio-economics. Population density (figure 3*a*) has a high vulnerability in the northeastern part of the country and along the coast, and is low in the central and eastern parts of the country. Vulnerability for children under 5 years (figure 3*b*) follows a similar trend. Groundwater dependence for domestic use (figure 3*c*) shows a high vulnerability in the northern and western parts of South Africa, as well as the interior. These are areas where surface water availability is limited and are also the areas where agriculture has traditionally been practised (figure 3*d*). The vulnerability for percentage of female-headed households is high in the northern parts and along the east coast of South Africa. The aggregated map indicates a medium vulnerability for most parts of the country, with some areas of low vulnerability in the northwestern parts of South Africa, and high vulnerability areas in some of the northeastern parts of the country.

#### 3.2.2. Overlay maps

Overlays of Astronomy Assessment Areas (protection areas for the Square Kilometer Array and Southern African Large Telescope (A. Tiplady 2012, personal communication)), subterranean groundwater control areas [31], areas where prospecting and mining, as well as petroleum exploration and production is legally prohibited (S. Holness 2013, personal communication), roads [32] and TCP and ER areas [33] were included on the socio-economic vulnerability map.

## 4. Conclusion

In terms of developing vulnerability maps with the vulnerability indicators ‘surface water’, ‘groundwater’, ‘vegetation’, ‘seismicity’ and ‘socio-economics’, similar aspects would also warrant protection in countries that plan to embark on UOG extraction, or where UOG extraction is already performed [3,34]. However, the unique features in South Africa in terms of complex geology where dolerite dykes intruded the native country rocks [11,35], the complex fractured rock aquifer systems [11,36] and the limited water availability [34,37] warrant a cautious approach regarding water resources in our endeavour to extract UOG, because the environmental and socio-economic consequences linked to impacts of UOG extraction on water resources may be much more severe than in other countries where UOG is currently extracted. As the mapping methodology is based on a comparison of more vulnerable areas with less vulnerable areas, it does not prescribe the absolute content of specific indicators. Different countries may derive different levels of vulnerability based on their judgement of the levels of threat represented by UOG extraction, and depending on their local circumstances. Comparing one country's vulnerability assessment with another country's vulnerability assessment is therefore not recommended.

The interactive vulnerability map that was developed for South Africa is important for spatial planning and may help the government and practitioners in performing strategic environmental assessments, as well as assessing licence applications and EIAs, because relevant vulnerability information is available in a central location and presented in a comprehensible format. Decision-makers usually struggle with making decisions on licences because all the relevant information is not easily accessible in one central location. International experts also commented on the need to have a centralized database for UOG extraction and its related activities. Konschnik & Dayalu [38] also stress that it is important that public disclosure of fracking data be enhanced to ensure that policymakers, researchers, industry and other stakeholders have access to comprehensive and reliable information on the localities of active and abandoned wells as well as related data that are important for the protection of natural resources and human health.

It is important to note that areas that are classified as low vulnerability on this interactive map should not be interpreted as areas that will experience no direct, indirect or residual impact from UOG extraction. The map does therefore not suggest that UOG extraction will be perfectly acceptable in low sensitivity areas and that these areas are ‘freed up’ for unopposed development. Rather these areas should be viewed as areas where UOG may potentially be extracted, based on the outcome of detailed environmental impact assessments and accompanying environmental management programmes aimed at identifying and ensuring the achievement of impact avoidance or mitigation.

Lastly, the indicators used in this map and any similar maps that indicate vulnerability to UOG extraction for other countries should be reviewed on a regular basis. This will ensure that the most relevant indicators that are available at any given moment are used for these vulnerability maps.

## Data accessibility

The source code for the interactive vulnerability map is available from the following website: https://fracking.webmaps.co.za/. The map data are available from the Dryad Digital Repository: http://dx.doi.org/10.5061/dryad.2sv13 [39].
